# Modulation of *Tcf7l2* Expression Alters Behavior in Mice

**DOI:** 10.1371/journal.pone.0026897

**Published:** 2011-10-27

**Authors:** Daniel Savic, Margaret G. Distler, Greta Sokoloff, Nancy A. Shanahan, Stephanie C. Dulawa, Abraham A. Palmer, Marcelo A. Nobrega

**Affiliations:** 1 Department of Human Genetics, University of Chicago, Chicago, Illinois, United States of America; 2 Committee on Molecular Pathogenesis and Molecular Medicine, University of Chicago, Chicago, Illinois, United States of America; 3 Department of Psychiatry and Behavioral Neuroscience, University of Chicago, Chicago, Illinois, United States of America; University of Illinois at Chicago, United States of America

## Abstract

The comorbidity of type 2 diabetes (T2D) with several psychiatric diseases is well established. While environmental factors may partially account for these co-occurrences, common genetic susceptibilities could also be implicated in the confluence of these diseases. In support of shared genetic burdens, *TCF7L2*, the strongest genetic determinant for T2D risk in the human population, has been recently implicated in schizophrenia (SCZ) risk, suggesting that this may be one of many loci that pleiotropically influence both diseases. To investigate whether *Tcf7l2* is involved in behavioral phenotypes in addition to its roles in glucose metabolism, we conducted several behavioral tests in mice with null alleles of *Tcf7l2* or overexpressing *Tcf7l2*. We identified a role for *Tcf7l2* in anxiety-like behavior and a dose-dependent effect of *Tcf7l2* alleles on fear learning. None of the mutant mice showed differences in prepulse inhibition (PPI), which is a well-established endophenotype for SCZ. These results show that *Tcf7l2* alters behavior in mice. Importantly, these differences are observed prior to the onset of detectable glucose metabolism abnormalities. Whether these differences are related to human anxiety-disorders or schizophrenia remains to be determined. These animal models have the potential to elucidate the molecular basis of psychiatric comorbidities in diabetes and should therefore be studied further.

## Introduction

An expanding body of evidence in the literature documents an increased co-occurrence of type 2 diabetes (T2D) with psychiatric disorders [1], [2], [3], [4], [5], [6], [7], [8], [9], [10], [11], [12]. However, the nature of the underlying co-morbidity is unclear. While one explanation for this confluence of seemingly unrelated disorders are environmental or socioeconomic factors such as diet and access to health care, an alternative possibility is shared genetic susceptibilities independently leading to an increased risk of both T2D and psychiatric disorders.

A potential role for common genetic factors is clearly documented in the historical comorbidity between T2D and schizophrenia (SCZ) [13], [14]. Though environmental factors, chief among them the use of antipsychotic medications and in particular atypical antipsychotic drugs, have been utilized to explain the increased risk of T2D through weight gain [15], the reported comorbidity between SCZ and T2D predates the use of antipsychotics [16], [17], [18], [19], [20]. In support of these early observations, several recent studies have highlighted an enhanced predisposition to diabetes in drug naïve schizophrenics [21], [22], [23], [24], [25]. A putative shared genetic burden is further evidenced through an increased prevalence of T2D in families affected by schizophrenia [26], [27] as well as through linkage studies for SCZ and T2D that have identified several overlapping genomic intervals [13]. Taken together, these data support the possible existence of genetic factors that mediate the co-occurrence of T2D with other psychiatric disorders.

A recent association study implicated variation within the Transcription factor 7-like 2 (*TCF7L2*) gene locus with schizophrenia risk [28]. Importantly, variation in *TCF7L2* was previously associated with T2D risk and is the strongest genetic determinant for T2D in human populations [29], [30], [31], Given these genetic associations, *TCF7L2* could serve as a key regulator of both glucose homeostasis and behavior and consequently represents an ideal candidate for interrogating the potential role of common genetic underpinnings in the comorbidity between T2D and psychiatric disorders.

We have recently developed a *Tcf7l2* null allele in mice, and simultaneously generated transgenic mice overexpressing *Tcf7l2* [32]. Our previous analyses defined a direct role for *Tcf7l2* on glucose tolerance and consequently susceptibility to T2D [32]. Here we examined behavior phenotypes in our *Tcf7l2* mouse models in the open field, light dark box, fear conditioning and prepulse inhibition.

## Methods

### Ethics Statement

All mice were housed at the University of Chicago. Veterinary care was available on a 24-hour basis. Mice were monitored daily for any signs of illness or discomfort. All experiments were conducted in strict accordance with institutional rules and approved by the University of Chicago Institutional Animal Care and Use Committee, protocol number 71607 (A.A.P.).

### Generation of Tcf7l2 knockout and BAC transgenic mice

Tcf7l2 knockout mice were generated using zinc finger nucleases while *Tcf7l2* overexpressing mice were generated using BAC recombineering as previously described [32]. All three *Tcf7l2* knockout lines and only heterozygous null mice (*Tcf7l2* ^+/−^) were used for testing as homozygous null mice displayed perinatal lethality. Wild-type littermates were utilized as controls for behavioral testing. Male mice were used for behavioral testing. Genotyping was conducted as previously outlined [32].

### Mouse husbandry

All mice were housed in cages (maximum of 5 animals per cage) with free access to food and water. Animals at 6–7 weeks of age were transferred for behavioral testing. After transfer, mice were allowed to acclimate to the new environment for at least 1 week prior to testing. For all behavioral tests, mice were transferred from the vivarium in home cages to the testing room and allowed to acclimate for 30 min prior to testing.

### Open field testing

The open field consisted of a 40×40×40 cm Plexiglas chamber under bright illumination (∼300 lux). After acclimation, mice were placed into the center (20×20 cm) of the open field. For 5 minutes, behavior was recorded and automatically scored using Ethovision XT (Noldus, Wageningen, Netherlands).

### Light dark box testing

The light-dark box apparatus consisted of the open field chamber described above, bisected by a black Plexiglas insert (20×40×40 cm) with a 4×4 cm door that allowed mice to explore both dark and light areas. After acclimation, mice were placed in the light compartment, and activity was monitored for 5 minutes. Behavior was recorded and automatically scored using Ethovision XT (Noldus, Wageningen, Netherlands).

### Fear conditioning

Fear conditioning was a 3-day paradigm and has previously been described in detail [33]. Fear conditioning occurred in standard conditioning chambers (29×19×25 cm with a stainless steel floor grid; Med Associates, St. Albans, VT, USA) with a light on the top of the chamber providing dim illuminations (∼3 lux). Each chamber was housed within a sound-attenuated chamber with a vent fan on one wall providing masking of background noise in the testing room. Freezing was digitally recorded and analyzed with FreezeFrame software (Actimetrics, Wilmette, IL, USA).

Day 1: Mice were placed in the test chamber (MedAssociates), and baseline freezing was measured. Mice were exposed to a tone twice (30 seconds, 85 dB, 3 kHz) that co-terminated with a footshock (2 seconds, 0.5 mA). A 30-second interval separated the two tone-footshock pairings.

Day 2: Test conditions were identical to day 1, but neither tone nor footshocks were presented.

Day 3: The chamber was altered in several ways: a different experimenter wore a different style of gloves; the transfer cages had no bedding; the metal shock grid was covered with a gray plastic floor; a bent gray plastic wall was inserted into the test chamber; a yellow light filter was placed over the chamber lights; chambers were cleaned with 0.1% acetic acid solution; and the vent fan was partially obstructed to change the background noise. The tone was presented at the same times as on day 1, but no foot shock was administered.

### Prepulse Inhibition

Startle chambers consisted of nonrestrictive Plexiglas cylinders 5 cm in diameter resting on a Plexiglas platform in a ventilated chamber (San Diego Instruments, San Diego, CA) as described elsewhere [34]. Sixty-five consecutive 1-ms readings were recorded beginning at startle stimulus onset to obtain the amplitude of the animals' startle response to each stimulus [34]. Sound levels were measured as described elsewhere using the A weighting scale [35]. For each test session, mice were exposed to five different trials: a 40-msec broadband 120 dB burst (Pulse Alone trial); three different Prepulse + Pulse trials in which either 20-msec long 3 dB, 6 dB, or 12 dB above background stimuli preceded the120 dB pulse by 100 msec (onset to onset); and a No Stimulus trial, in which only background noise (65 dB) was presented. Trials were presented in a pseudo-random order and separated by an average of 15 s (range: 9–20 s). The test session began with a 5-min acclimation period, which was followed by four blocks of test trials. Blocks one and four consisted of six consecutive Pulse Alone trials, while blocks two and three each contained six Pulse Alone trials, five of each kind of Prepulse + Pulse trial, and four No Stimulus trials. Prepulse inhibition (PPI) was calculated as [100 – (Prepulse—Pulse trial/averaged Pulse Alone)×100]. Pulse Alone values were calculated as the mean of startle values from blocks two and three. ANOVAs with genotype as a between-subjects factor, and block and prepulse intensity as within-subject factors were applied to averaged PPI values. For startle reactivity, ANOVAs with genotype as a between-subjects factor was applied to averaged Pulse Alone values from blocks two and three to determine whether any effects of these variables on startle confounded the interpretation of PPI results.

### Statistical tests

Data are shown as mean ± standard error of the mean (S.E.M). An unpaired two-sided Student's t-test was utilized to test for significance in the open field, light dark box and fear conditioning. For PPI, a repeated measures ANOVA was used with PPI intensity as the repeated measure and genotype as single factor.

## Results

### Open field Testing

**Figure 1:**
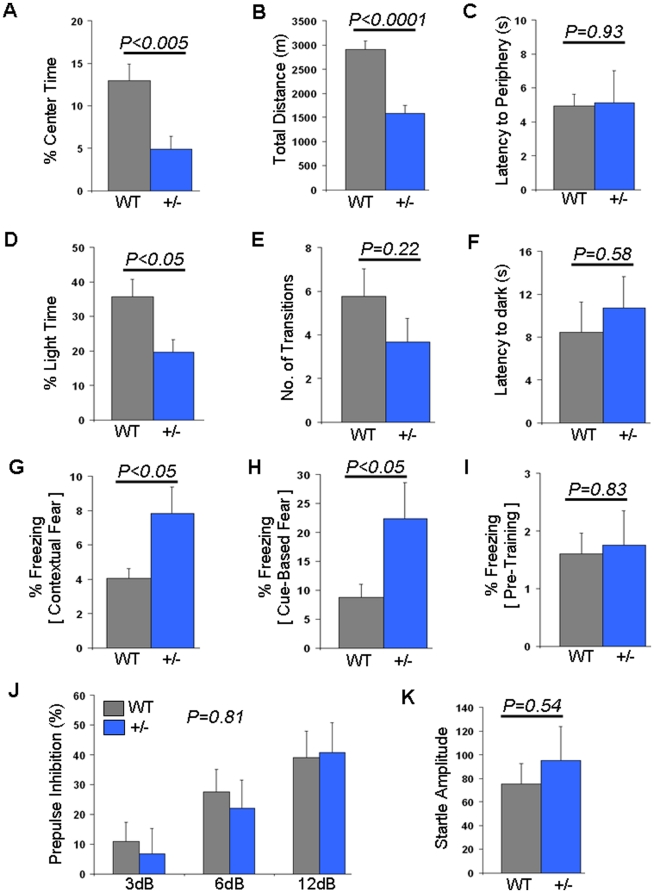
Behavioral analyses from Tcf7l2 ablation. (A)–(C) Open field testing. (A) Center time shown as a percent of total time (%) in wild-type (WT, n = 14) and heterozygous null (+/−, n = 12) mice. (B) Total distance shown in meters (m) in wild-type (WT, n = 14) and heterozygous null (+/−, n = 12) mice. (C) Latency to periphery shown in seconds (s) in wild-type (WT, n = 14) and heterozygous null (+/−, n = 12) mice. (D)–(F) Light dark box testing. (D) Time in light shown as percent of total time (%) in wild-type (WT, n = 14) and heterozygous null (+/–, n = 12) mice. (E) Number of transitions between the light and dark compartments in wild-type (WT, n = 14) and heterozygous null (+/–, n = 12) mice. (F) Latency to the dark compartment shown in seconds (s) in wild-type (WT, n = 14) and heterozygous null (+/–, n = 12) mice. (G)–(I) Fear conditioning. (G) Contextual fear shown as a percent time spent freezing (%) in wild-type (WT, n = 14) and heterozygous null (+/–, n = 12) mice. (H) Cue-based fear shown as a percent time spent freezing (%) in wild-type (WT, n = 14) and heterozygous null (+/–, n = 12) mice. (I) Pre-training freezing shown as a percent time spent freezing (%) in wild-type (WT, n = 14) and heterozygous null (+/–, n = 12) mice. (J) Prepulse inhibition in wild-type (WT, n = 14) and heterozygous null (+/–, n = 12) mice. Prepulse inhibition (%) using prepulses of 3, 6 and 12 decibels (dB) are shown. (K) Startle response in wild-type (WT, n = 14) and heterozygous null (+/–, n = 12) mice. Wild-type data are shown in gray while heterozygous null data are shown in blue.

To elucidate a role for *Tcf7l2* in anxiety-like behavior, adult heterozygous null mice (*Tcf7l2* ^+/−^) were first tested in the open field. *Tcf7l2* ^+/−^ mice exhibited decreased time in the center of the arena compared to wild-type littermates (P<0.005; Figure 1A). Analysis of total distance between knockout mice and their wild-type littermates identified a significant decrease in locomotor activity (p<0.0001; Figure 1B). Further, latency to periphery was not significantly different between the two groups of mice (p = 0.93; Figure 1C).

**Figure 2:**
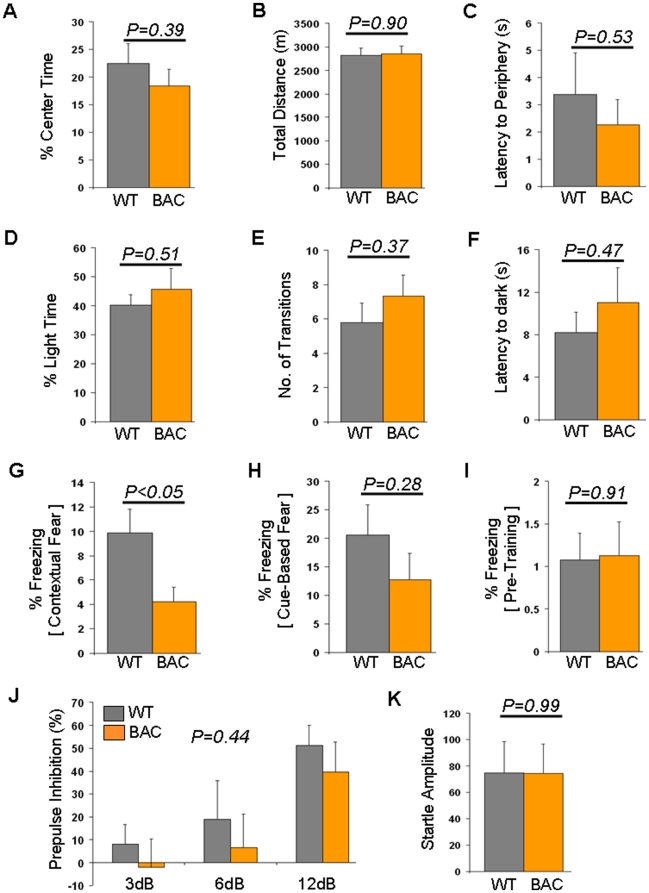
Behavioral analyses from *Tcf7l2* overexpression. (A)–(C) Open field testing. (A) Center time shown as a percent of total time (%) in wild-type (WT, n = 9) and BAC transgenic (BAC, n = 9) mice. (B) Total distance shown in meters (m) in wild-type (WT, n = 9) and BAC transgenic (BAC, n = 9) mice. (C) Latency to periphery shown in seconds (s) in wild-type (WT, n = 9) and BAC transgenic (BAC, n = 9) mice. (D)–(F) Light dark box testing. (D) Time in light shown as a percent of total time (%) in wild-type (WT, n = 9) and BAC transgenic (BAC, n = 9) mice. (E) Number of transitions between the light and dark compartments in wild-type (WT, n = 9) and BAC transgenic (BAC, n = 9) mice. (F) Latency to the dark compartment shown in seconds (s) in wild-type (WT, n = 9) and BAC transgenic (BAC, n = 9) mice. (G) – (I) Fear conditioning. (G) Contextual fear shown as a percent time spent freezing (%) in wild-type (WT, n = 9) and BAC transgenic (BAC, n = 9) mice. (H) Cue-based fear shown as a percent time spent freezing (%) in wild-type (WT, n = 9) and BAC transgenic (BAC, n = 9) mice. (I) Pre-training freezing shown as a percent time spent freezing (%) in wild-type (WT, n = 9) and BAC transgenic (BAC, n = 9) mice. (J) Prepulse inhibition in wild-type (WT, n = 9) and BAC transgenic (BAC, n = 9) mice. Prepulse inhibition (%) using prepulses of 3, 6 and 12 decibels (dB) are shown. (K) Startle response in wild-type (WT, n = 9) and BAC transgenic (BAC, n = 9) mice. Wild-type data are shown in gray while BAC transgenic data are shown in orange.

During open-field testing, adult BAC transgenic mice overexpressing *Tcf7l2* did not show significant differences in center time (p = 0.39; Figure 2A), locomotor activity (p = 0.90; Figure 2B), or latency to periphery (p = 0.53; Figure 2C) compared to wild-type littermates.

### Light dark box testing

To further investigate the role of *Tcf7l2* in anxiety-like behavior we tested mice in the light dark box. *Tcf7l2* ^+/−^ mice spent significantly less time in the light compartment compared to their wild-type littermates (p<0.05; Figure 1D). *Tcf7l2* ^+/−^ mice did not differ from their wild-type litter mates in the number of transitions between the light and dark compartments (p = 0.22; Figure 1E) and latency to dark was not significantly different between the two groups of animals (p = 0.58; Figure 1F).

We next tested BAC transgenic mice in the light dark box. Similar to the open field test results, we did not observe significant differences in time spent in the light compartment (p = 0.51; Figure 2D). Further, no significant differences were detected in the number of transitions between the light and dark compartments (p = 0.37; Figure 2E) or the latency to transition to the dark compartment (p = 0.47; Figure 2F).

### Fear Conditioning

We also explored fear conditioning in the *Tcf7l2* ^+/−^ mice. Fear conditioning measures learned fear and is correlated with differences in anxiety-like behavior [33]. *Tcf7l2* ^+/−^ mice displayed increased freezing to both context (p<0.05; Figure 1G) and tone (p<0.05; Figure 1H). Importantly, pre-training freezing was not significantly different between the two groups (p = 0.83; Figure 1I), suggesting that alterations in generalized freezing behavior were not responsible for the observed differences.

BAC transgenic mice subsequently underwent fear conditioning. Surprisingly, we identified a reciprocal phenotype in our BAC transgenic mice compared to the *Tcf7l2* ^+/−^ mice. Overexpression of *Tcf7l2* significantly decreased freezing to context (p = 0.05, Figure 2G). Freezing to the tone was not significantly different between BAC transgenic mice and wild-type littermates (p = 0.28, Figure 2H). This suggests that *Tcf7l2* overexpression increases contextual, but not cue-based fear. Importantly, pre-training freezing was not significantly different between the two groups (p = 0.91, Figure 2I), indicating that baseline differences in freezing do not confound the results.

### Prepulse Inhibition

There were no differences in prepulse inhibition (PPI) (Figures 1J and 2J). Importantly, both genotypes showed increasing levels of PPI as the prepulse intensities increased, demonstrating that the startle response in these animals was effectively modulated by the prepulses. Startle responses between animals were not significantly different (Figures 1K and 2K).

## Discussion

To functionally test the hypothesis that the observed co-morbidity between T2D and psychiatric disorders might be partially due to pleiotropic effects of *TCF7L2* alleles, we performed several behavioral studies using mice with null alleles of *Tcf7l2* as well as mice over-expressing *Tcf7l2*. The use of these mouse models allowed for the testing of a range of *Tcf7l2* copy numbers that might be similar to gain- or loss-of-function alleles in humans. Our data support a role for *Tcf7l2* in behavior, thereby demonstrating that this transcription factor can influence a diverse set of processes.

Using the open field and light dark box tests, we observed anxiety-like phenotypes in *Tcf7l2* ^+/−^ mice; ablation of Tcf7l2 led to decreased time in the center of the arena and in the light compartment. However, these phenotypes were coincident with differences in locomotor activity, namely decreased distance traveled in the open field. While this may confound the interpretation of anxiety phenotypes, we did not observe significant differences in latency to periphery in open field testing. Both latency to the dark compartment and the number of transitions were not significant in light dark box testing, suggesting this test might not have been confounded by differences in activity. Taken together, these observations support a role for *Tcf7l2* in anxiety-like behaviors; future studies of the impact of human alleles of *TCF7L2* on anxiety are warranted.

Our mouse model demonstrated a dose-dependent role for *Tcf7l2* in fear learning. Ablation of *Tcf7l2* led to enhanced fear learning while mice overexpressing *Tcf7l2* displayed an impairment of fear learning. Interestingly, this gene-dosage effect was only observed for contextual fear, which is believed to be a hippocampus-dependent process [36] and therefore may have some relevance for SCZ. Indeed, disruptions of contextual processing are known to occur in SCZ [37] and this endophenotype is routinely used in rodent and human studies [38], [39].

While our study uncovered a role for *Tcf7l2* on anxiety and fear learning, we did not identify significant differences in PPI in both heterozygous null mice and BAC transgenic mice. This is significant because PPI is a behavioral test that can be more directly related to the observation of increased risk for SCZ in humans [28]. However, we cannot exclude potential differences in PPI in older mice as behavioral studies were conducted on mice 7–8 weeks of age at the start of behavioral testing.

Importantly, none of the mice were diabetic during behavioral testing as we previously reported glucose intolerance in mice overexpressing *Tcf7l2* only after a high fat diet stress [32]. As a result, this enabled the dissociation of diabetes-related secondary effects in this study. In light of this, our study design is able to detect independent genetic effects on behavior, which is a significant advantage over studies of co-morbidity in humans. Our design allows us to conclude that Tcf7l2 directly regulates behavior.

The idea that seemingly disparate diseases may result from shared susceptibility loci is intriguing and reflects the integrated nature of our genome. Indeed, *TCF7L2* functions as a transcriptional regulator of the canonical Wnt signaling pathway that maintains broad roles in development regulating cell fate, survival and proliferation [40], [41], [42]. Genes such as *TCF7L2* that are involved in pathways that harbor pleiotropic functions represent strong candidates in this respect. Our study demonstrates that *TCF7L2* carries pleiotropic effects and provides added evidence of possible common genetic underpinning that may explain the historical comorbidities between T2D and psychiatric disorders.

## References

[1] Huang CJ, Chiu HC, Lee MH, Wang SY (2011). Prevalence and incidence of anxiety disorders in diabetic patients: a national population-based cohort study.. Gen Hosp Psychiatry.

[2] Anderson RJ, Freedland KE, Clouse RE, Lustman PJ (2001). The prevalence of comorbid depression in adults with diabetes: a meta-analysis.. Diabetes Care.

[3] Mukherjee S, Decina P, Bocola V, Saraceni F, Scapicchio PL (1996). Diabetes mellitus in schizophrenic patients.. Compr Psychiatry.

[4] Dixon L, Weiden P, Delahanty J, Goldberg R, Postrado L (2000). Prevalence and correlates of diabetes in national schizophrenia samples.. Schizophr Bull.

[5] Regenold WT, Thapar RK, Marano C, Gavirneni S, Kondapavuluru PV (2002). Increased prevalence of type 2 diabetes mellitus among psychiatric inpatients with bipolar I affective and schizoaffective disorders independent of psychotropic drug use.. J Affect Disord.

[6] Subramaniam M, Chong SA, Pek E (2003). Diabetes mellitus and impaired glucose tolerance in patients with schizophrenia.. Can J Psychiatry.

[7] Collins MM, Corcoran P, Perry IJ (2009). Anxiety and depression symptoms in patients with diabetes.. Diabet Med.

[8] Grigsby AB, Anderson RJ, Freedland KE, Clouse RE, Lustman PJ (2002). Prevalence of anxiety in adults with diabetes: a systematic review.. J Psychosom Res.

[9] Egede LE, Zheng D, Simpson K (2002). Comorbid depression is associated with increased health care use and expenditures in individuals with diabetes.. Diabetes Care.

[10] Khuwaja AK, Lalani S, Dhanani R, Azam IS, Rafique G (2010). Anxiety and depression among outpatients with type 2 diabetes: A multi-centre study of prevalence and associated factors.. Diabetol Metab Syndr.

[11] Medved V, Jovanovic N, Knapic VP (2009). The comorbidity of diabetes mellitus and psychiatric disorders.. Psychiatr Danub.

[12] Bouwman V, Adriaanse MC, van 't Riet E, Snoek FJ, Dekker JM (2010). Depression, anxiety and glucose metabolism in the general dutch population: the new Hoorn study.. PLoS One.

[13] Gough SC, O'Donovan MC (2005). Clustering of metabolic comorbidity in schizophrenia: a genetic contribution?. J Psychopharmacol.

[14] Lin PI, Shuldiner AR (2010). Rethinking the genetic basis for comorbidity of schizophrenia and type 2 diabetes.. Schizophr Res.

[15] Holt RI, Peveler RC, Byrne CD (2004). Schizophrenia, the metabolic syndrome and diabetes.. Diabet Med.

[16] Lorenz WF (1922). Sugar tolerance in dementia praecox and other mental disorders.. Arch Neurol Psychiatry.

[17] Kasanin J (1926). The blood sugar curve in mental disease, II: the schizophrenic (dementia praecox) group.. Arch Neurol Psychiatry.

[18] Braceland FJ, Meduna LJ, Vaichulis JA (1945). Delayed action of insulin in schizophrenia.. American Journal of Psychiatry.

[19] Freeman H (1946). Resistance to insulin in mentally disturbed soldiers.. Arch Neurol Psychiatry.

[20] Kooy FH (1919). Hyperglycemia in mental disorders.. Brain.

[21] Ryan MC, Collins P, Thakore JH (2003). Impaired fasting glucose tolerance in first-episode, drug-naive patients with schizophrenia.. Am J Psychiatry.

[22] Cohn TA, Remington G, Zipursky RB, Azad A, Connolly P (2006). Insulin resistance and adiponectin levels in drug-free patients with schizophrenia: A preliminary report.. Can J Psychiatry.

[23] Spelman LM, Walsh PI, Sharifi N, Collins P, Thakore JH (2007). Impaired glucose tolerance in first-episode drug-naive patients with schizophrenia.. Diabet Med.

[24] Dasgupta A, Singh OP, Rout JK, Saha T, Mandal S (2010). Insulin resistance and metabolic profile in antipsychotic naive schizophrenia patients.. Prog Neuropsychopharmacol Biol Psychiatry.

[25] Guest PC, Wang L, Harris LW, Burling K, Levin Y (2010). Increased levels of circulating insulin-related peptides in first-onset, antipsychotic naive schizophrenia patients.. Mol Psychiatry.

[26] Fernandez-Egea E, Miller B, Bernardo M, Donner T, Kirkpatrick B (2008). Parental history of type 2 diabetes in patients with nonaffective psychosis.. Schizophr Res.

[27] Mukherjee S, Schnur DB, Reddy R (1989). Family history of type 2 diabetes in schizophrenic patients.. Lancet.

[28] Hansen T, Ingason A, Djurovic S, Melle I, Fenger M (2011). At-Risk Variant in TCF7L2 for Type II Diabetes Increases Risk of Schizophrenia.. Biol Psychiatry.

[29] Lyssenko V (2008). The transcription factor 7-like 2 gene and increased risk of type 2 diabetes: an update.. Curr Opin Clin Nutr Metab Care.

[30] Cauchi S, El Achhab Y, Choquet H, Dina C, Krempler F (2007). TCF7L2 is reproducibly associated with type 2 diabetes in various ethnic groups: a global meta-analysis.. J Mol Med.

[31] Grant SF, Thorleifsson G, Reynisdottir I, Benediktsson R, Manolescu A (2006). Variant of transcription factor 7-like 2 (TCF7L2) gene confers risk of type 2 diabetes.. Nat Genet.

[32] Savic D, Ye H, Aneas I, Park SY, Bell GI (2011). Alterations in TCF7L2 expression define its role as a key regulator of glucose metabolism..

[33] Ponder CA, Kliethermes CL, Drew MR, Muller J, Das K (2007). Selection for contextual fear conditioning affects anxiety-like behaviors and gene expression.. Genes Brain Behav.

[34] Dulawa SC, Geyer MA (1996). Psychopharmacology of prepulse inhibition in mice.. Chin J Physiol.

[35] Mansbach RS, Geyer MA, Braff DL (1988). Dopaminergic stimulation disrupts sensorimotor gating in the rat.. Psychopharmacology (Berl).

[36] Marschner A, Kalisch R, Vervliet B, Vansteenwegen D, Buchel C (2008). Dissociable roles for the hippocampus and the amygdala in human cued versus context fear conditioning.. J Neurosci.

[37] Hemsley DR (2005). The development of a cognitive model of schizophrenia: placing it in context.. Neurosci Biobehav Rev.

[38] Balu DT, Carlson GC, Talbot K, Kazi H, Hill-Smith TE (2010). Akt1 deficiency in schizophrenia and impairment of hippocampal plasticity and function..

[39] Pohlack ST, Nees F, Ruttorf M, Witt SH, Nieratschker V (2011). Risk variant for schizophrenia in the neurogranin gene impacts on hippocampus activation during contextual fear conditioning..

[40] MacDonald BT, Tamai K, He X (2009). Wnt/beta-catenin signaling: components, mechanisms, and diseases.. Dev Cell.

[41] Clevers H (2006). Wnt/beta-catenin signaling in development and disease.. Cell.

[42] Moon RT, Kohn AD, De Ferrari GV, Kaykas A (2004). WNT and beta-catenin signalling: diseases and therapies.. Nat Rev Genet.

